# Serum ferritin to predict efficacy of adult HLH induction therapy

**DOI:** 10.1080/07853890.2022.2081870

**Published:** 2022-05-28

**Authors:** Philip T. Murphy, Philip W. Murphy

**Affiliations:** Department of Haematology, Beaumont Hospital, Dublin, Ireland

Regarding “Serum ferritin is a good indicator for predicting the efficacy of adult HLH induction therapy” recently published in Annals of Medicine, Hua et al. retrospectively analysed the clinical data of 102 adult patients with hemophagocytic lymphohistiocytosis (HLH) admitted to their hospital between January and December 2017, based on HLH-2004 diagnostic criteria [1]. The authors report that serum ferritin at 2–4 weeks post induction can predict the efficacy of induction therapy for adult HLH. The annual incidence of HLH has been estimated at 1.04 in 1,000,000 in China in 2016 [2], with a roughly similar estimate of 1 in 800,000 per year in a Japanese survey [3]. It is, therefore, somewhat surprising that Hua et al. diagnosed this condition in over 100 patients in their hospital in just one calendar year. The possibility of overdiagnosis of HLH in this study needs to be considered, especially as a local NK cell activity assay was used as part of diagnostic criteria and it is unclear if there has been reproducibility of this assay by other groups. It would certainly be interesting to know the mean number of diagnostic criteria fulfilled by the 102 patients, as well as the percentage of these patients who had low or absent NK-cell activity according to the local assay. Hua and colleagues could also have stratified their cases according to the HLH predictive scoring system (HScore) proposed by Fardet et al. in 2014 [4] before correlating with serum ferritin and efficacy of therapy.

## Author contributions

PTM was involved in conception, drafting of paper and final approval of the version to be published. PWM was involved in revising the article for intellectual content and final approval of the version to be published. All authors agree to be accountable for all aspects of the work.

## Data Availability

Data that support this article are available within the article.
